# Evolutionary dynamics and research hotspots of phage applications against *Klebsiella pneumoniae* infections from the past to the new era

**DOI:** 10.3389/fmicb.2026.1906882

**Published:** 2026-08-19

**Authors:** Jiawei Zhang, Yanan Liu, Yaru Wang, Wei Zhang, Jianhua Liu, Zhihua Zhang

**Affiliations:** 1 Department of Postgraduate, Hebei North University, Zhangjiakou, China; 2 Hebei Key Laboratory of Pathogenic Mechanisms and Diagnosis & Treatment Technologies for Lung Microbiome, The First Affiliated Hospital of Hebei North University, Zhangjiakou, Hebei, China

**Keywords:** bibliometric analysis, CiteSpace, *Klebsiella pneumonia*, phage, VOSviewer

## Abstract

**Background:**

*Klebsiella pneumoniae* is a critical opportunistic pathogen, with carbapenem-resistant *K. pneumoniae* and hypervirulent *K. pneumoniae* posing severe threats to global public health due to escalating antimicrobial resistance. As natural bacterial predators, phages represent a highly promising alternative to conventional antibiotics. The aim of this study was to systematically evaluate the research landscape, collaborative networks, and evolutionary trends of phage applications against *Klebsiella pneumoniae* infections through bibliometric analysis.

**Methods:**

Core bibliographic data were retrieved from the Web of Science Core Collection, Scopus, and PubMed databases. Following rigorous literature screening based on predefined inclusion and exclusion criteria, mainstream bibliometric analysis tools, including VOSviewer and CiteSpace, were comprehensively deployed to map and analyze the temporal dynamics of publication outputs, international collaboration networks among countries, institutions, authors, and journals, reference co-citation matrices, and keyword co-occurrence configurations, alongside timeline visualizations and citation burst detection, thereby systematically delineating the evolutionary blueprint of this research domain.

**Results:**

A total of 1,275 valid original publications were ultimately enrolled. Chronologically, the global annual publication volume exhibited a pronounced exponential growth trajectory (*y* = 3.4589⋅*e*^0.2381(*x*−2007)^, *R*^2^ = 0.9706). Geographically and structurally, China ranked first globally in research productivity with 242 documents (18.98%), the Chinese Academy of Sciences (49 documents) and investigator Jianqiang Li (30 documents) were identified as the most prolific institution and core author, respectively. Regarding publishing channels, Frontiers in Microbiology dominated in both publication scale (55 documents) and network integration density (TLS = 237). Research hotspot evolution indicated a profound paradigm shift, where contemporary citation bursts are significantly dominated by “phage resistance” (strength = 3.34) and “lytic activity” (strength = 2.77), with both frontiers remaining actively sustained into 2026.

**Conclusion:**

Over the past two decades, research has expanded exponentially, with the knowledge architecture transitioning from descriptive biological characterization to deep genetic mechanisms and engineering-driven design. Future efforts should prioritize elucidating the molecular mechanisms of phage resistance, optimizing lytic enzyme efficacy, and accelerating multicenter translational interventions to combat multi-drug resistant *K. pneumoniae*.

## 1 Introduction

*Klebsiella pneumoniae* (KP) is a critical Gram-negative opportunistic pathogen designated as a critical-priority pathogen on the 2024 WHO Bacterial Priority Pathogens List, and serves as a primary cause of nosocomial infections (Paczosa and Mecsas, 2016; Aggarwal et al., 2024; World Health Organization, 2024). The pathogen typically colonizes the human respiratory, gastrointestinal, and urogenital tracts, and can induce fatal conditions such as pneumonia, urinary tract infections, meningitis, and bacteremia (Podschun and Ullmann, 1998; Paczosa and Mecsas, 2016). Based on its virulence and pathogenic characteristics, KP is categorized into “classic” (cKP) and “hypervirulent” (hvKP) pathotypes (Shon et al., 2013; Paczosa and Mecsas, 2016). While cKP predominantly targets immunocompromised patients in hospital settings, hvKP is capable of causing severe invasive infections even in healthy individuals, including pyogenic liver abscesses, endophthalmitis, and necrotizing fasciitis (Shon et al., 2013; Paczosa and Mecsas, 2016; Zhang et al., 2016). Recent evidence warns that the convergent evolution of multidrug resistance and hypervirulence is driving the emergence of “superbugs” that possess both characteristics, posing a dire threat to global public health (Zhang et al., 2016; Arcari and Carattoli, 2023).

The rapid escalation of antimicrobial resistance has severely constrained the clinical management of KP infections. Notably, carbapenem-resistant *K. pneumoniae* (CRKP) has been designated as a top-priority pathogen by the World Health Organization, necessitating the urgent development of novel antibacterial strategies (Tacconelli et al., 2018; Aggarwal et al., 2024). While polymyxins and tigecycline represent the last line of defense for treating CRKP, the annual detection rate of resistant strains continues to climb, and their clinical utility is frequently limited by significant nephrotoxicity and suboptimal bactericidal activity (Tacconelli et al., 2018; Petrosillo et al., 2019). Furthermore, the ability of KP to form robust biofilms creates a physical barrier that prevents effective antibiotic penetration (Bassetti et al., 2018; Guerra et al., 2022). Given the protracted timelines and high failure rates associated with discovering truly novel antibiotic classes, the exploration of alternative therapeutic modalities with unique mechanisms of action has become an imperative in infectious disease research (Rios et al., 2016; Bassetti et al., 2018; Aggarwal et al., 2024).

As natural bacterial predators, phages represent a highly promising antimicrobial strategy due to their profound host specificity, ability to self-replicate at the site of infection, and effective lysis of multidrug-resistant strains (Chanishvili, 2012; Hatfull et al., 2022). Current intervention paradigms have transitioned from monotherapy to diversified modalities: phage cocktails suppress the emergence of resistant variants by targeting multiple host receptors (Martins et al., 2022; Le et al., 2023), while phage-antibiotic synergy has demonstrated substantial potential for enhancing therapeutic efficacy across various infection states (Nir-Paz et al., 2019; Luong et al., 2020). Furthermore, phage-derived enzymes offer a novel paradigm for biofilm eradication and host resistance inhibition by precisely degrading KP capsular polysaccharides (CPS) and cleaving the bacterial cell wall (Latka et al., 2017; Cheetham et al., 2024). Concurrently, de novo genomic engineering driven by artificial intelligence (AI) is accelerating the clinical translation of precision phage therapeutics (Xing et al., 2025a; Brixi et al., 2026).

Despite the exponential expansion of both fundamental and clinical research concerning KP phages, the scientific landscape remains fragmented. Existing systematic reviews predominantly focus on molecular and biological mechanisms (Caflisch et al., 2019; Wang et al., 2023), leaving a conspicuous gap in the quantitative assessment of global production patterns, disciplinary evolution, and research hotspots over the past two decades. Bibliometric analysis represents a mathematical and statistical methodology based on public literature databases, capable of elucidating collaborative networks among countries, institutions, and authors, while forecasting emerging scientific growth poles (Zhang et al., 2020). This study aims to utilize VOSviewer and CiteSpace visualization tools to systematically examine the developmental trajectory of global KP phage applications from January 1, 2007, to March 14, 2026. Our objective is to provide evidence-based insights for optimizing clinical treatment protocols, identifying critical technological barriers, and facilitating the strategic allocation of international scientific resources.

## 2 Methods

### 2.1 Search strategy and material

A systematic and comprehensive literature retrieval was executed across three primary electronic databases: the Web of Science Core Collection (WoSCC), Scopus, and PubMed, targeting publications indexed from January 1, 2007, to March 14, 2026. To accommodate database-specific syntaxes, the standardized search string was constructed using Boolean operators as follows: (“*Klebsiella pneumoniae*” OR “*K. pneumoniae*” OR “CRKP” OR “hvKP” OR “MDR-KP”) AND (“Phage*” OR “Bacteriophage*”). The document type was strictly restricted to peer-reviewed original research articles published in English. All comprehensive metadata, including full records and cited references, were exported in standardized programmatic formats to facilitate downstream computational bibliometric analysis.

### 2.2 Data extraction and eligibility criteria

**FIGURE 1:**
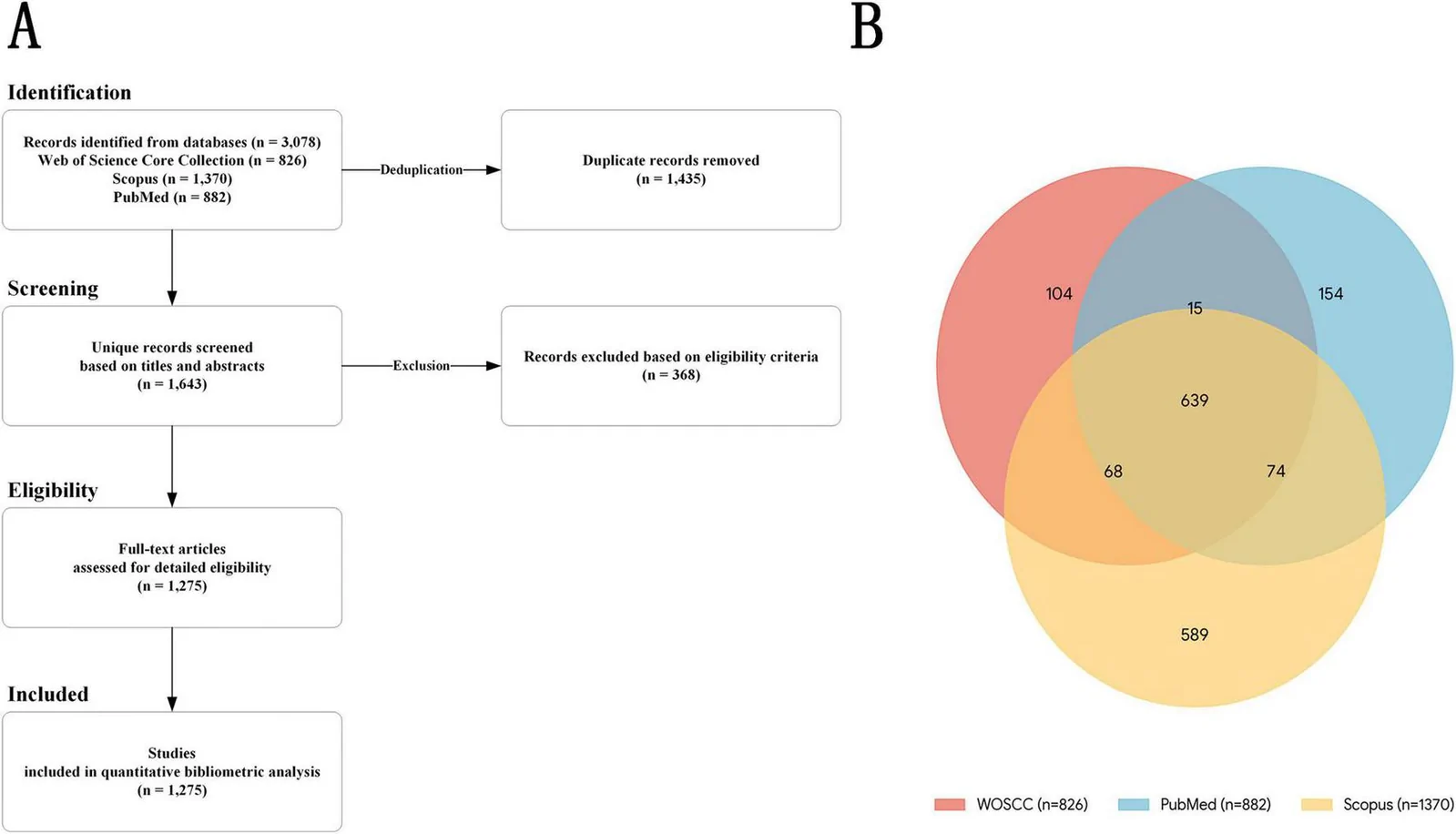
Flowchart of the literature screening process **(A)** and Venn diagram of the eligible literature after deduplication across the three databases **(B)**. The total volume of eliminated duplicates (*n* = 1,435) is mathematically calculated as: (15 + 68 + 74) + 2 × 639 = 1,435, where dual-database overlap records are removed once and the central triple-database overlap records (*n* = 639) are removed twice to retain 1,643 unique documents.

To ensure methodological transparency, literature identification and screening protocols were performed in general accordance with the PRISMA-ScR guidelines. The initial search yielded a crude pool of 3,078 records, comprising 826 from WoSCC, 1,370 from Scopus, and 882 from PubMed (Figure 1A). To mitigate the methodological opacity inherent in merging multi-source datasets, bibliographic records derived from Scopus and PubMed were programmatically converted into the standardized Web of Science (WoS) format utilizing the built-in Data Converter in CiteSpace (version 6.1.R1). The converted datasets were subsequently consolidated with the primary WoSCC records, and 1,435 duplicate entries were systematically eliminated using the CiteSpace deduplication engine. This programmatic filtering pipeline secured a clean pool of 1,643 unique publications. The distinct contribution and intersection of literature across the three designated databases are explicitly quantified and visualized via the Venn diagram in Figure 1B.

Following data cleansing, a rigorous two-stage screening protocol was independently performed by three investigators (JZ, YL, and YW). Titles and abstracts were evaluated against predefined inclusion criteria governed by a modified PICOS framework optimized for broad bibliometric profiling: Target subjects (P): KP isolates, infected cellular models, animal models, or clinical patients, Interventions (I): therapeutic applications of phages or phage-derived lytic enzymes, Study design (S): primary clinical trials and laboratory-based experimental research. Comparators (C) and Outcomes (O) were not restricted within this eligibility framework. This deliberate omission was designed to maximize retrieval sensitivity and secure exhaustive coverage of the mechanistic, genomic, and evolutionary landscapes of phage research, thereby preventing the premature exclusion of non-comparative basic library studies. Review articles, conference abstracts, editorial materials, and non-English publications were excluded. Discrepancies during the screening phase were adjudicated via consensus. After excluding 368 records unrelated to the core topic, a finalized dataset comprising 1,275 research articles was secured for subsequent knowledge domain mapping (Figure 1A).

### 2.3 Bibliometric and visualized analysis

To systematically analyze literature trends and research hotspots in the domain of phage applications against *Klebsiella pneumoniae* infections, this study deployed multiple bibliometric software programs for comprehensive quantitative analysis and scientific knowledge mapping. Microsoft Office Excel was utilized to compile, organize, and model annual publication volumes and temporal growth trajectories. VOSviewer (version 1.6.18) is a bibliometric analysis software that can extract the key information from numerous publications (van Eck and Waltman, 2010), which is often used to build collaboration, co-citation and co-occurrence networks (Pan et al., 2018; Yeung and Mozos, 2020). In our study, the software mainly completes the following analysis: country, institution and author analysis, journal and co-cited references analysis, and all keyword co-occurrence analysis. Within these networks, the intensity of collaborative partnerships and structural integration was operationalized using Total Link Strength (TLS), which quantifies the cumulative weight of link pathways connecting a focal node to all other entities in the system. Additionally, Citations per Publication (CPP) was employed to evaluate the academic impact and citation density of these entities, operationalized as the ratio of cumulative citations to the total number of published documents. Furthermore, in the network visualization maps generated by VOSviewer, visual elements are rigorously standardized to represent specific bibliometric parameters. Specifically, the node size is proportional to the occurrence frequency or publication volume of a given item, while the thickness of the connecting link lines explicitly represents the strength of collaboration, co-occurrence, or co-citation between the nodes. Additionally, distinct color coding is applied to designate discrete modules automatically partitioned by the software’s built-in modularity clustering algorithm, where nodes sharing the identical color denote tightly integrated thematic, collaborative, or intellectual networks. CiteSpace (version 6.1.R1) is another software developed by Professors Chen C for bibliometric analysis and visualization (Synnestvedt et al., 2005; Pan et al., 2018). Through the application of CiteSpace’s specialized algorithmic functions, timeline visualizations were generated to partition the mapped dataset into distinct thematic clusters based on log-likelihood ratios, while citation burst detection was executed to identify high-impact reference nodes and emerging keywords experiencing sharp increases in citation intensity over specified periods. Through the synergistic application of these multi-dimensional methodologies, this research dynamically elucidates the developmental trajectories, structural cross-linking, and contemporary research frontiers of anti-*Klebsiella pneumoniae* phage therapeutics.

## 3 Results

### 3.1 Publication trends

**FIGURE 2:**
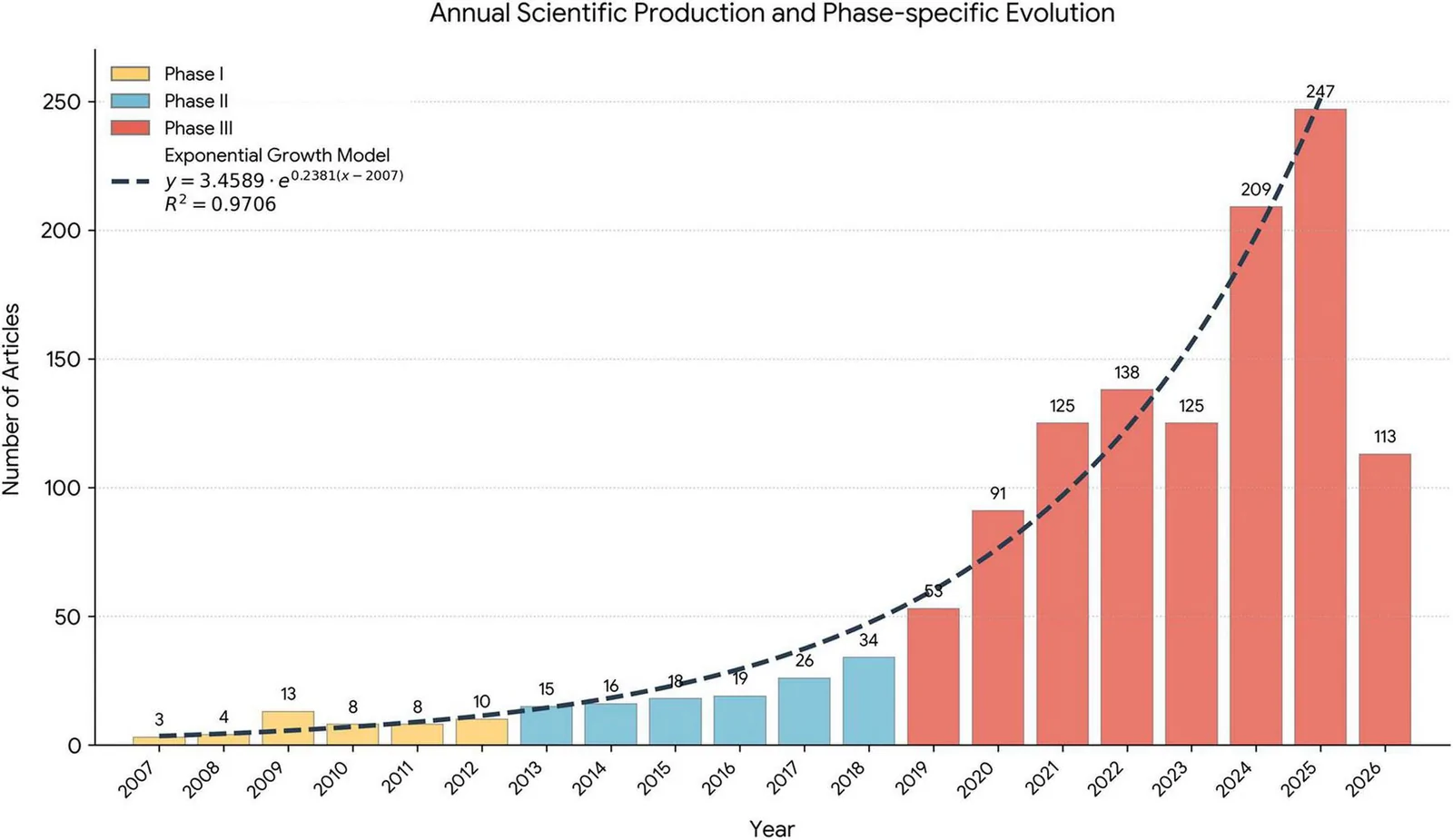
The global annual number of publications on phage applications against *Klebsiella pneumoniae* infections.


A total of 1,275 records from 2007 to May 14, 2026 were extracted (Figure 2). Annual publication volume was analyzed. Due to incomplete data for 2026, the prediction model was constructed using the 2007–2025 datasets. The growth trajectory was fitted using an exponential regression model:

$$y=3.4589\cdot e^{0.2381\,\left(x-2007\right)}\left(R^{2}=0.9706\right).$$

The development of phage applications against KP infections was divided into three phases. In Phase I from 2007 to 2012, annual publications ranged from 3 in 2007 to 13 in 2009, totaling 46 papers and representing 3.61% of the dataset. In Phase II from 2013 to 2018, annual output rose from 15 to 34, with intervening counts of 16, 18, 19, and 26, totaling 128 publications and remaining below 50 papers per year. In Phase III from 2019 to 2025, annual publications increased from 53 to 247, representing a 4.6-fold increase.

### 3.2 Countries/regions analysis

**FIGURE 3:**
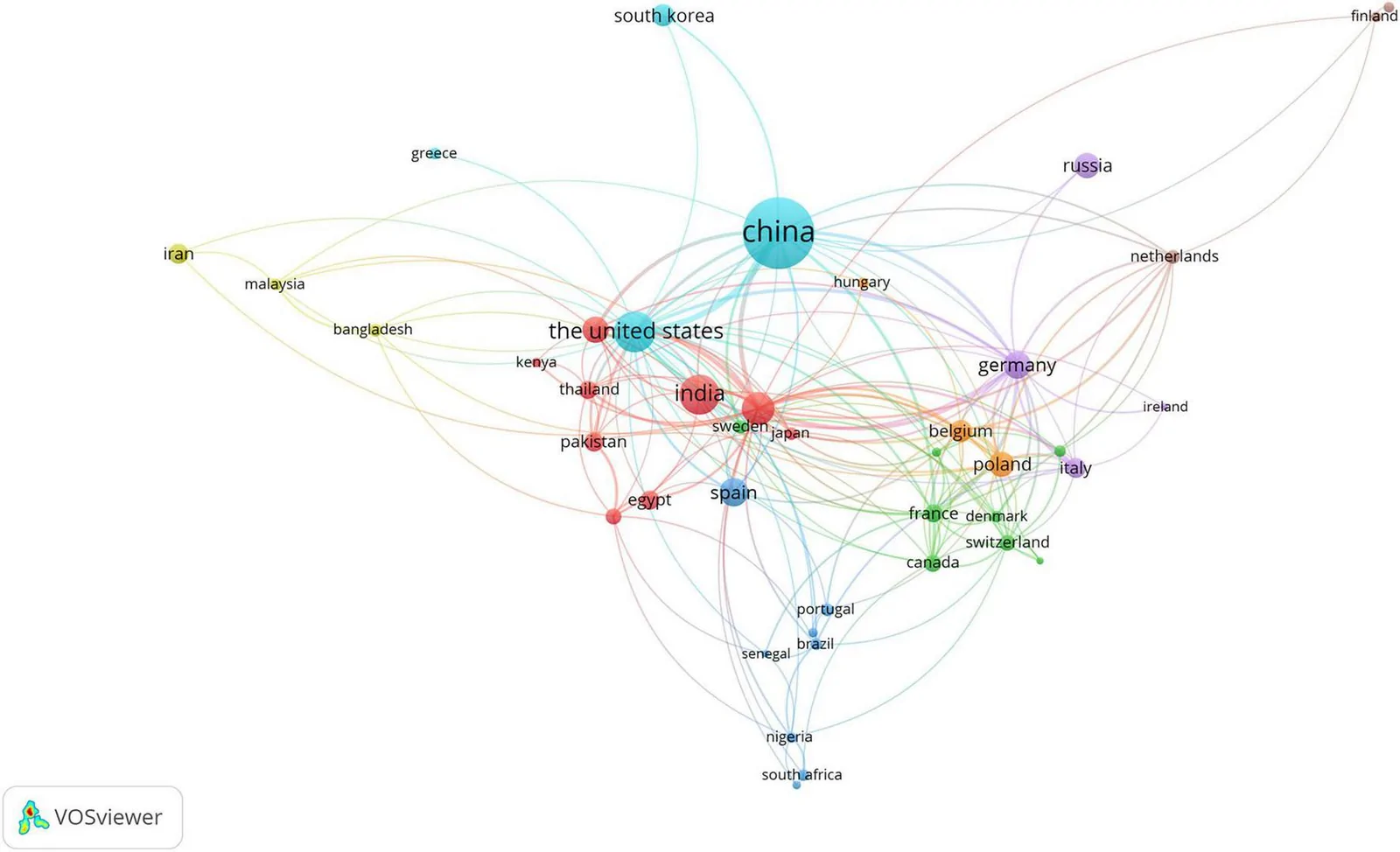
The cluster visualization of countries/regions on phage applications against *Klebsiella pneumoniae* infections. Distinct color coding designates different collaborative networks automatically partitioned by the algorithm; nodes sharing the same color represent tightly cross-linked geopolitical research alliances.

The spatial distribution of research was analyzed using the top 10 productive nations and their co-authorship matrix. The top 10 countries contributed 644 publications, representing 50.51% of the total dataset (*N* = 1,275). In Table 1, China ranked first with 242 documents (18.98%). The United States (*n* = 80, 6.27%) and India (*n* = 75, 5.88%) ranked second and third. Output for the remaining nations ranged from 25 to 51 publications. Publications were clustered across Asia, North America, and Europe. Although Asian nations led in publication volume, their CPP were lower than those of European and Oceanian nations. China and India recorded CPP values of 0.26 and 1.19. Russia had the highest CPP at 179.39. Germany and Australia recorded CPP values of 30.56 and 20.32, respectively. A cooperation network with a minimum threshold of 3 publications per node yielded 43 countries (Figure 3). Total Link Strength (TLS) quantified network connectivity. China (TLS = 48), the United Kingdom (TLS = 41), and the United States (TLS = 41) were the primary hubs. Germany (TLS = 30) and Australia (TLS = 26) showed moderate connectivity. Despite its high CPP, Russia recorded a TLS of 3. South Korea also recorded a TLS of 3.

**TABLE 1:** The top 10 most productive countries/regions on phage applications against *Klebsiella pneumoniae* infections.

Rank	Country (continent)	Documents	Percentage (%)	Citations	CPP	TLS
1	China (Asia)	242	18.98%	63	0.26	48
2	The United States (North America)	80	6.27%	225	2.81	41
3	India (Asia)	75	5.88%	89	1.19	16
4	The United Kingdom (Europe)	51	4.00%	320	6.27	41
5	Spain (Europe)	39	3.06%	255	6.54	7
6	Germany (Europe)	36	2.82%	1,100	30.56	30
7	Australia (Oceania)	34	2.67%	691	20.32	26
8	Poland (Europe)	31	2.43%	515	16.61	13
9	Russia (Europe/Asia)	31	2.43%	5,561	179.39	3
10	South Korea (Asia)	25	1.96%	17	0.68	3

CPP, Citations per Publication; TLS, Total Link Strength.

### 3.3 Institution analysis

**FIGURE 4:**
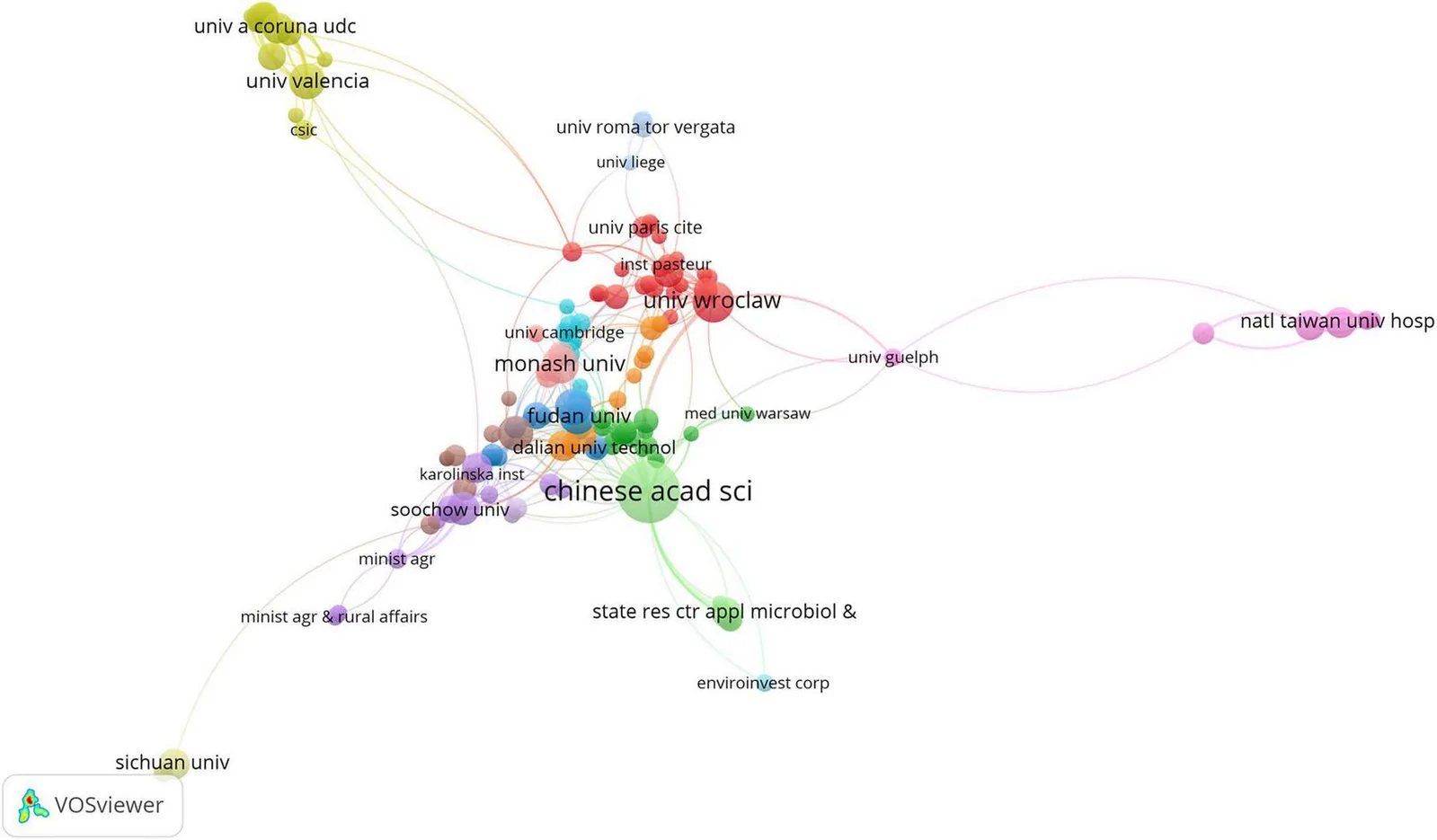
The cluster visualization of institutions on phage applications against *Klebsiella pneumoniae* infections. The color coding partitions the institutions into distinct research clusters based on network density; institutions within the same colored group demonstrate integrated regional or thematic collaborative networks.

Institutional publication profiles, citation trajectories, and co-authorship matrices were analyzed. In Table 2, the top 10 institutions contributed 202 publications (15.84%). Chinese Academy of Sciencess published 49 documents, followed by Panjab University with 26 and the University of Wrocław with 21. Chinese institutions constituted 50% of the top 10. Zhejiang University ranked eighth in publications (*n* = 15) with a CPP of 19.33. Shanghai Jiao Tong University, Fudan University, and the University of A Coruña recorded CPP values of 11.38, 10.50, and 10.33, respectively. The University of Wrocław and the Chinese Academy of Sciences recorded CPP values of 1.19 and 3.08. A co-authorship network with a minimum threshold of 3 publications per node comprised 182 entities (Figure 4). Chinese Academy of Sciencess (TLS = 11), Shanghai Jiao Tong University (TLS = 8), and the University of Wrocław (TLS = 8) were the primary hubs. Nanjing Agricultural University and Panjab University recorded TLS values of 2 and 1.

**TABLE 2:** The top 10 most productive institutions on phage applications against *Klebsiella pneumoniae* infections.

Rank	Organization (country)	Documents	Percentage (%)	Citations	CPP	TLS
1	Chinese Academy of Sciences (China)	49	3.84%	151	3.08	11
2	Panjab University (India)	26	2.04%	90	3.46	1
3	University of Wrocław (Poland)	21	1.65%	25	1.19	8
4	Monash University (Australia)	17	1.33%	54	3.18	3
5	Shanghai Jiao Tong University (China)	16	1.25%	182	11.38	8
6	Fudan University (China)	16	1.25%	168	10.5	7
7	University of Valencia (Spain)	16	1.25%	157	9.81	3
8	Zhejiang University (China)	15	1.18%	290	19.33	5
9	Nanjing Agricultural University (China)	14	1.10%	39	2.79	2
10	University of A Coruña (Spain)	12	0.94%	124	10.33	3

CPP, Citations per Publication; TLS, Total Link Strength.

### 3.4 Author analysis

**FIGURE 5:**
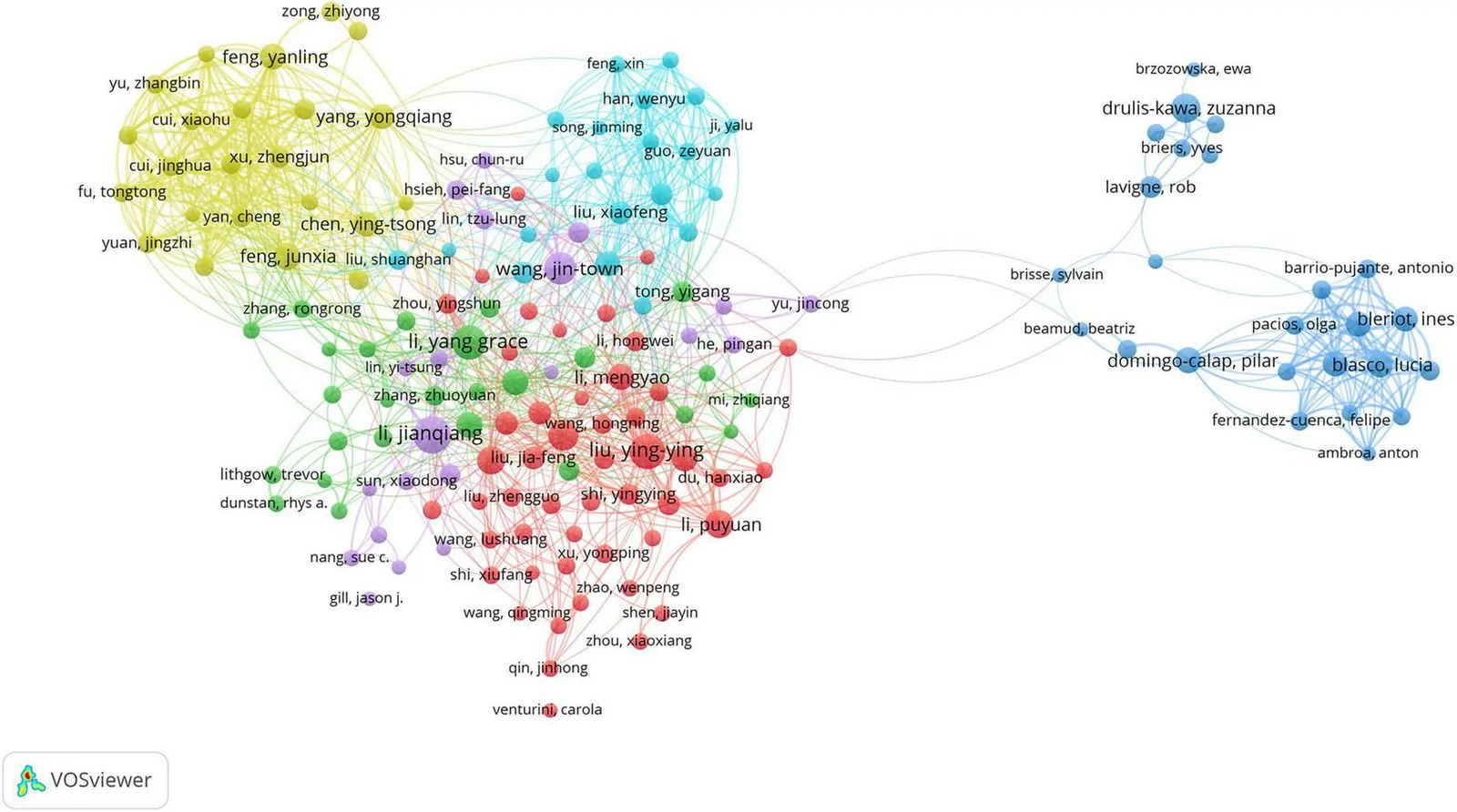
The co-authorship cluster visualization mapping the core investigator cohort. The distinct color coding identifies specialized research teams or scientific schools of thought; researchers within the same colored block represent a tightly connected academic community.

Author publication performance, citation trajectories, and co-authorship networks were analyzed. In Table 3, the top 10 authors contributed 220 publications (17.25% of the total dataset). Jianqiang Li published the most documents with 30 papers. Sanjay Chhibber and Ying-Ying Liu each published 27 documents. Output for the remaining authors ranged from 16 to 25 publications. Yangheng Zhang (*n* = 17) and Jin-Town Wang (*n* = 23) each recorded 305 total citations. Yangheng Zhang recorded the highest CPP at 17.94, followed by Jin-Town Wang (CPP = 13.26) and Puyuan Li (CPP = 12.00). Ying-Ying Liu and Sanjay Chhibber recorded CPP values of 2.56 and 1.48, respectively. A co-authorship network with a minimum threshold of 5 publications per node identified 186 researchers (Figure 5). Ying-Ying Liu (TLS = 17), Jianqiang Li (TLS = 13), and Xiang-Min Li (TLS = 12) were the primary hubs. Sanjay Chhibber and Zuzanna Drulis-Kawa each recorded a TLS of 2.

**TABLE 3:** Top 10 authors on phage applications against *Klebsiella pneumoniae* infections.

Rank	Author	Documents	Percentage (%)	Citations	CPP	TLS
1	Jianqiang Li	30	2.35%	181	6.03	13
2	Sanjay Chhibber	27	2.12%	40	1.48	2
3	Ying-Ying Liu	27	2.12%	69	2.56	17
4	Yang Grace Li	25	1.96%	151	6.04	11
5	Jin-Town Wang	23	1.80%	305	13.26	8
6	Xiang-Min Li	20	1.57%	84	4.2	12
7	Zuzanna Drulis-Kawa	18	1.41%	174	9.67	2
8	Yangheng Zhang	17	1.33%	305	17.94	9
9	Puyuan Li	17	1.33%	204	12	10
10	Junxia Feng	16	1.25%	173	10.81	2

CPP, Citations per Publication; TLS, Total Link Strength.

### 3.5 Journal analysis

**FIGURE 6:**
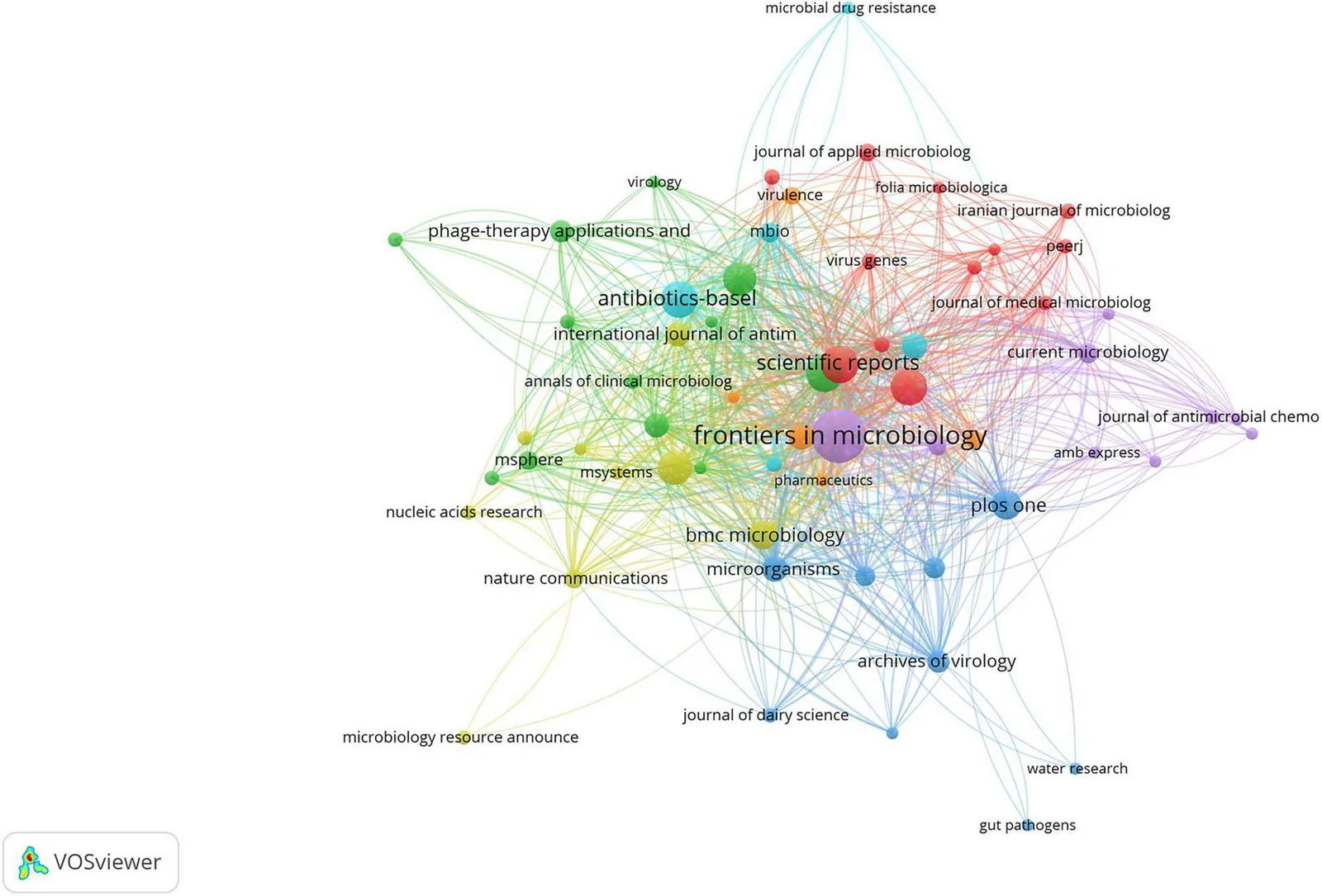
The citation network visualization of publishing venues and knowledge communication channels. The color coding differentiates major disciplinary or thematic citation clusters, grouping journals that frequently cross-cite each other or share similar intellectual trajectories into identical color blocks.

Journal publication scales, citation trajectories, and connectivity were evaluated. In Table 4, the top 10 journals contributed 247 papers (19.37% of the total dataset). Frontiers in Microbiology ranked first with 55 publications, followed by Scientific Reports (*n* = 27) and Viruses (*n* = 26). Microbial Pathogenesis (*n* = 25) and Antibiotics (*n* = 25) followed. Frontiers in Cellular and Infection Microbiology (*n* = 21) recorded the highest CPP at 25.14. Microbiology Spectrum (*n* = 22) and Frontiers in Microbiology (*n* = 55) recorded CPP values of 12.23 and 10.91, respectively. Antibiotics and Microorganisms recorded CPP values of 1.20 and 1.38, respectively. A citation network with a minimum threshold of 3 publications comprised 61 nodes (Figure 6). Frontiers in Microbiology recorded the highest TLS at 237. Scientific Reports (TLS = 136), Microbiology Spectrum (TLS = 116), and Viruses (TLS = 112) followed. Microorganisms (*n* = 13) recorded a TLS of 86. Microbial Pathogenesis (*n* = 25) recorded a TLS of 69.

**TABLE 4:** Top 10 journals on phage applications against *Klebsiella pneumoniae* infections.

Rank	Journal	Documents	Percentage (%)	Citations	CPP	TLS
1	Frontiers in microbiology	55	4.31%	600	10.91	237
2	Scientific reports	27	2.12%	54	2	136
3	Viruses	26	2.04%	270	10.38	112
4	Microbial pathogenesis	25	1.96%	216	8.64	69
5	Antibiotics	25	1.96%	30	1.2	79
6	Microbiology spectrum	22	1.73%	269	12.23	116
7	Frontiers in cellular and infection microbiology	21	1.65%	528	25.14	86
8	PLOS ONE	17	1.33%	61	3.59	63
9	BMC microbiology	16	1.25%	37	2.31	76
10	Microorganisms	13	1.02%	18	1.38	86

CPP, Citations per Publication; TLS, Total Link Strength.

### 3.6 Co-cited reference analysis

**FIGURE 7:**
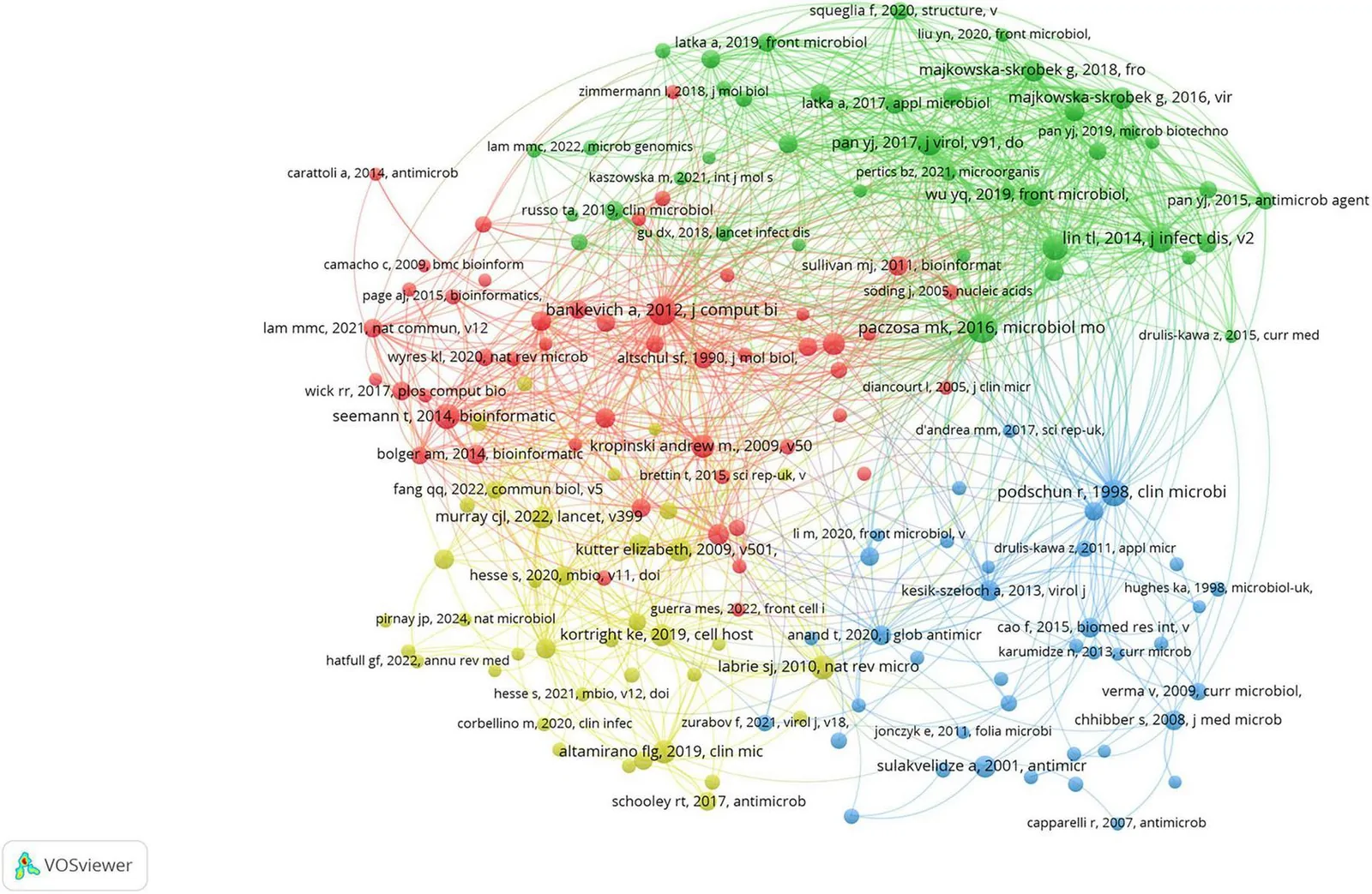
The reference co-citation network mapping the foundational knowledge base of phage applications against *Klebsiella pneumoniae* infections. The color coding categorizes the cited references into distinct clusters that represent specific foundational knowledge blocks or historic thematic paradigms.

Reference co-citation was analyzed. In Table 5, the top 20 co-cited references were compiled. Paczosa and Mecsas (2016) recorded 94 co-citations, followed by Bankevich et al. (2012) with 88 and Podschun and Ullmann (1998) with 79. Co-citation counts for the remaining top 20 references ranged from 43 to 75. Total Link Strength (TLS) quantified network connectivity (Figure 7). Pan et al. (2017) recorded the highest TLS at 317 (*n* = 72). Lin et al. (2014) and Paczosa and Mecsas (2016) each recorded a TLS of 308. Pires et al. (2016) recorded a TLS of 294 (*n* = 68). Majkowska-Skrobek et al. (2016), Bankevich et al. (2012), and Majkowska-Skrobek et al. (2018) recorded TLS values of 274, 272, and 271, respectively. Hsieh et al. (2017) and Wu et al. (2019) recorded TLS values of 244 and 227. Podschun and Ullmann (1998) recorded a TLS of 189. The remaining references in the top 20 cohort exhibited TLS values ranging from 113 to 186.

**TABLE 5:** Top 10 co-cited references on phage applications against *Klebsiella pneumoniae* infections.

Rank	Co-cited reference	Citations	TLS
1	Paczosa MK, 2016, Microbiol Mol Biol R, v80, p629, doi 10.1128/mmbr.00078-15	94	308
2	Bankevich A, 2012, J Comput Biol, v19, p455, doi 10.1089/cmb.2012.0021	88	272
3	Podschun R, 1998, Clin Microbiol Rev, v11, p589, doi 10.1128/cmr.11.4.589	79	189
4	Lin TL, 2014, J Infect Dis, v210, p1734, doi 10.1093/infdis/jiu332	75	308
5	Pan YJ, 2017, J Virol, v91, doi 10.1128/jvi.02457-16	72	317
6	Pires DP, 2016, Appl Microbiol Biot, v100, p2141, doi 10.1007/s00253-015-7247-0	68	294
7	Seemann T, 2014, Bioinformatics, v30, p2068, doi 10.1093/bioinformatics/btu153	68	158
8	Kropinski Andrew M., 2009, v501, p69, doi 10.1007/978-1-60327-164-6_7	58	163
9	Kutter Elizabeth, 2009, v501, p141, doi 10.1007/978-1-60327-164-6_14	57	159
10	Kortright KE, 2019, Cell Host Microbe, v25, p219, doi 10.1016/j.chom.2019.01.014	56	127
11	Majkowska-Skrobek G, 2018, Front Microbiol, v9, doi 10.3389/fmicb.2018.02517	55	271
12	Aziz RK, 2008, BMC Genomics, v9, doi 10.1186/1471-2164-9-75	54	164
13	Majkowska-Skrobek G, 2016, Viruses-Basel, v8, doi 10.3390/v8120324	53	274
14	Wu YQ, 2019, Front Microbiol, v10, doi 10.3389/fmicb.2019.02768	52	227
15	Kesik-Szeloch A, 2013, Virol J, v10, doi 10.1186/1743-422x-10-100	50	141
16	Eskenazi A, 2022, Nat Commun, v13, doi 10.1038/s41467-021-27656-z	46	113
17	Hsieh PF, 2017, Sci Rep-UK, v7, doi 10.1038/s41598-017-04644-2	46	244
18	Latka A, 2017, Appl Microbiol Biot, v101, p3103, doi 10.1007/s00253-017-8224-6	44	186
19	Nishimura Y, 2017, Bioinformatics, v33, p2379, doi 10.1093/bioinformatics/btx157	44	156
20	Moraru C, 2020, Viruses-Basel, v12, doi 10.3390/v12111268	43	155

TLS, Total Link Strength.

### 3.7 Keyword analysis

Keyword co-occurrence of Keywords Plus was analyzed. In Table 6, the top 20 keywords were compiled. “Phage” recorded 299 occurrences, followed by “other bacteria” (*n* = 183), “*klebsiella pneumoniae*” (*n* = 146), and “gene” (*n* = 140). Subsequent keywords included “infection” (*n* = 85), “resistance” (*n* = 70), “identification” (*n* = 66), “antibiotic resistance” (*n* = 66), and “virulence” (*n* = 65).

**TABLE 6:** Top 20 keywords on phage applications against *Klebsiella pneumoniae* infections.

Rank	Keyword plus	Occurrences	TLS	Rank	Keyword plus	Occurrences	TLS
1	Phage	299	571	11	Biofilm	52	127
2	Other bacteria	183	431	12	Mechanism	42	95
3	*Klebsiella pneumoniae*	146	376	13	Protein	41	96
4	Gene	140	343	14	Diversity	39	114
5	Infection	85	252	15	Epidemiology	38	115
6	Resistance	70	178	16	Polysaccharide	33	92
7	Identification	66	201	17	Evolution	30	80
8	Antibiotic resistance	66	179	18	Endolysin	24	49
9	Virulence	65	198	19	*Enterobacteriaceae*	21	64
10	Therapy	58	116	20	Bloodstream infection	21	57

TLS, Total Link Strength.

**FIGURE 8:**
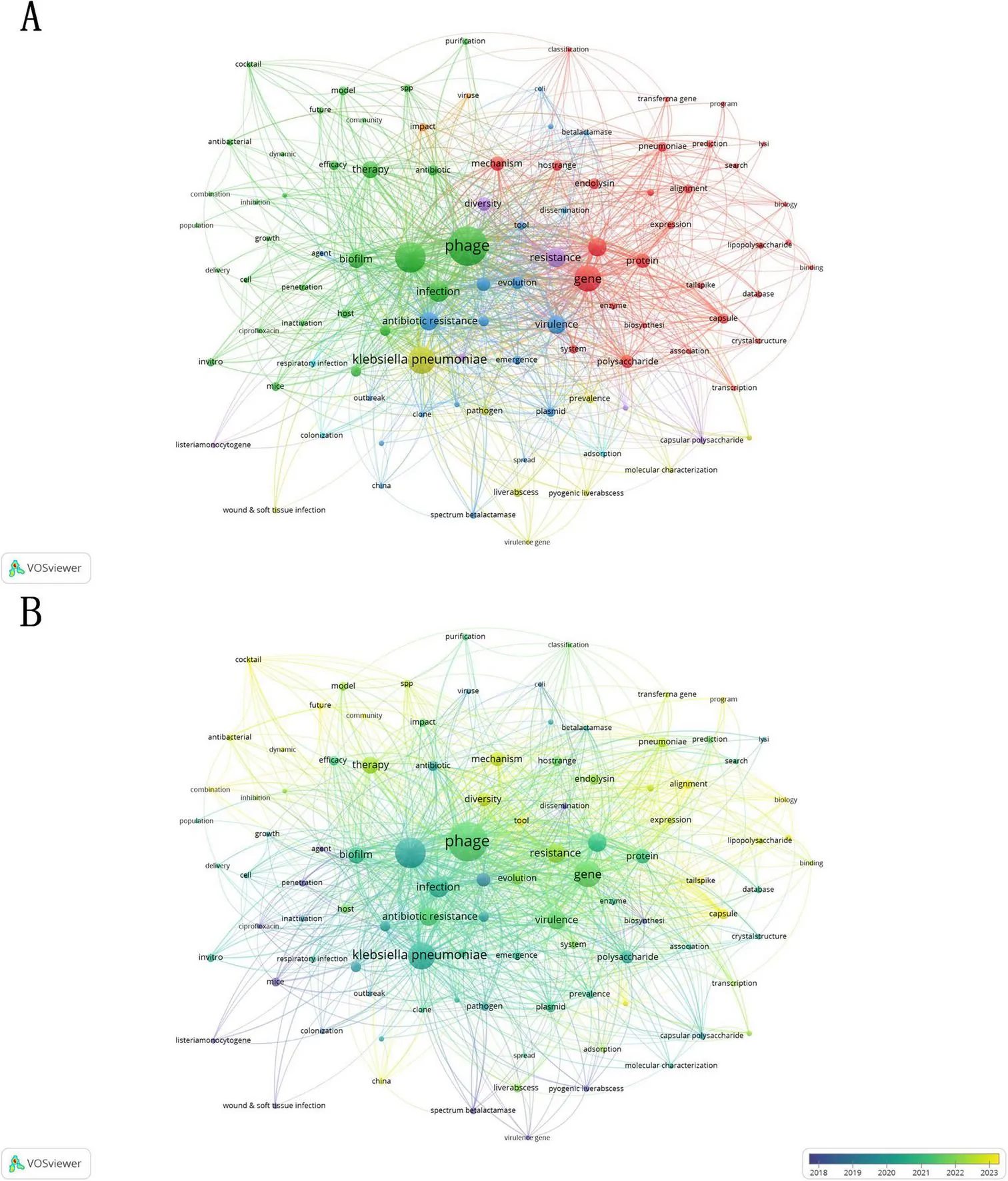
The scientific mapping of keyword co-occurrence for Keywords plus. **(A)** Network modularity cluster visualization view. The color coding explicitly delineates primary thematic modules calculated by the algorithm, where each distinct color represents a specific research domain or sub-field. **(B)** Overlay visualization view mapping the shifting frequency and temporal gradients.

The co-occurrence network yielded six color-coded modules (Figure 8A). The green cluster included phage, biofilm, therapy, and cocktail. The red cluster included gene, protein, endolysin, expression, and prediction. The blue cluster included antibiotic resistance, virulence, infection, and system. The yellow cluster included *klebsiella pneumoniae*, china, pathogen, and plasmid. The orange cluster included mechanism, hostrange, impact, and virus. The purple cluster included mice, respiratory infection, *Listeria monocytogenes*, and wound and soft tissue infection.

Shifting frequency and average publication years from 2018 to 2023 were mapped (Figure 8B). Keywords with the lowest average publication years were rendered in dark blue and purple. These included mice, respiratory infection, pyogenic liver abscess, *Listeria monocytogenes*, and wound and soft tissue infection. Terms with average publication years between 2020 and 2021 were rendered in green. These included biofilm, antibiotic resistance, phage, and therapy. Keywords with the highest recency up to 2023 were highlighted in yellow. These included tailspike, capsule, capsular polysaccharide, cocktail, and alignment.

**FIGURE 9:**
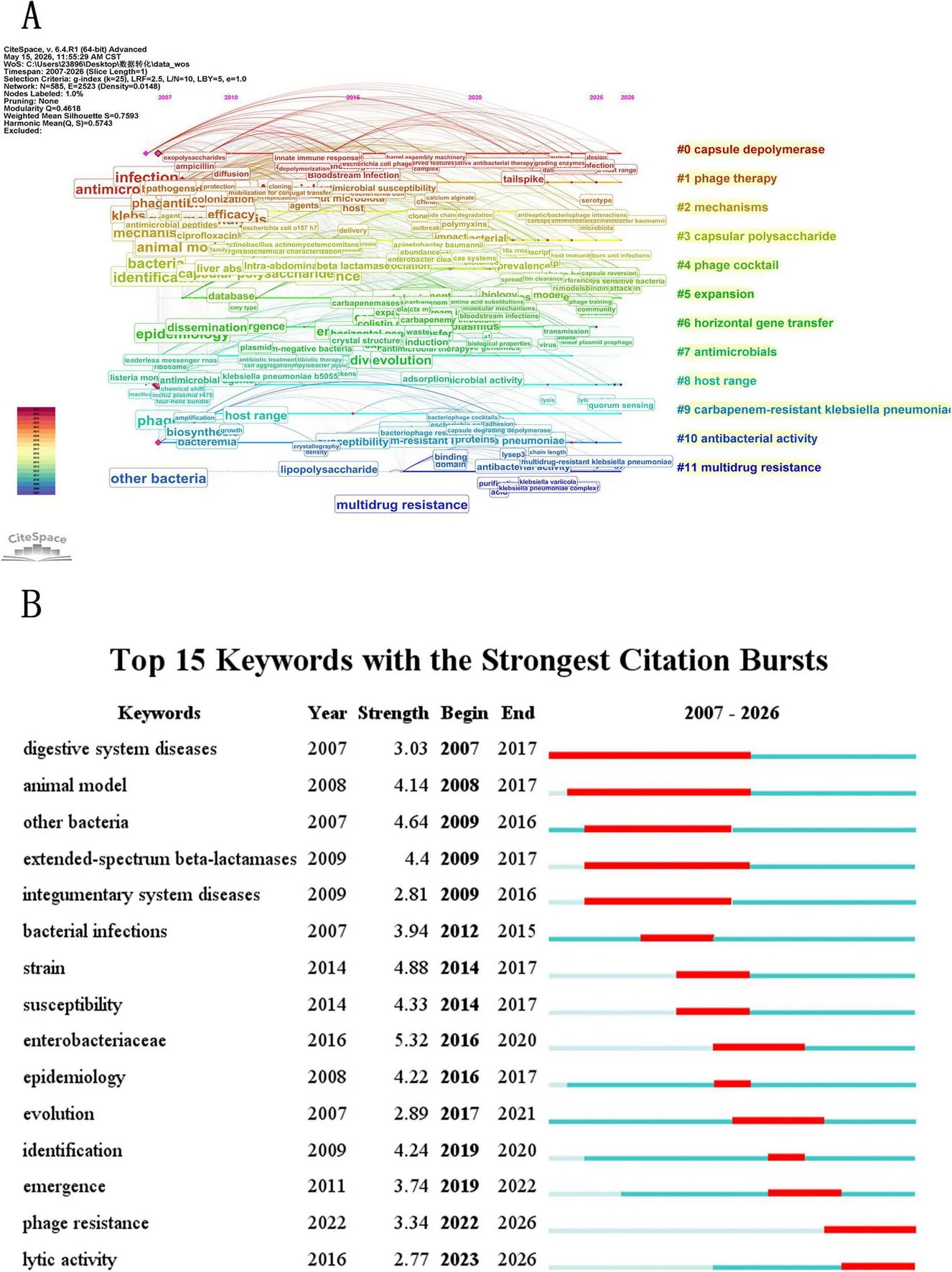
The timezone view **(A)** and keywords burst **(B)** on phage applications against *Klebsiella pneumoniae* infections.

CiteSpace was used to identify thematic subdivisions and intensity shifts. The timeline visualization partitioned the dataset into 12 thematic clusters (Figure 9A). These consisted of cluster 0 capsule depolymerase, cluster 1 phage therapy, cluster 2 mechanisms, cluster 3 capsular polysaccharide, cluster 4 phage cocktail, cluster 5 expansion, cluster 6 horizontal gene transfer, cluster 7 antimicrobials, cluster 8 host range, cluster 9 carbapenem-resistant *klebsiella pneumoniae*, cluster 10 antibacterial activity, and cluster 11 multidrug resistance. The top 15 keywords with citation bursts from 2007 to 2026 were logged (Figure 9B). “Digestive system diseases” had a citation burst from 2007 to 2017 with a strength of 3.03. “*Enterobacteriaceae*” recorded the maximum burst strength of 5.32 from 2016 to 2020. The most recent bursts were identified for “phage resistance” and “lytic activity.” “Phage resistance” burst from 2022 to 2026 with a strength of 3.34, and “lytic activity” burst from 2023 to 2026 with a strength of 2.77.

## 4 Discussion

### 4.1 The spatiotemporal landscape of global productivity

The bibliometric analysis reveals that a total of 1,275 valid bibliographic records concerning phage applications against KP infections were published between 2007 and May 2026. The chronological expansion of this academic domain was modeled using an exponential regression equation based on the completed annual datasets from 2007 to 2025. This growth trajectory demonstrated an exceptionally high goodness-of-fit (*R*^2^ = 0.9706), providing definitive empirical evidence that the field has undergone a powerful exponential expansion over the past two decades. Based on the fitted regression model, the projected annual publication volume for 2026 is estimated to reach approximately 319 papers. This projected zenith underscores that the current academic output is actively sustaining the rapid acceleration phase characteristic of the contemporary era, continuously attracting multi-dimensional scientific attention globally. Based on the empirical velocity of annual research production and chronological inflection points, the evolutionary trajectory of this domain can be systematically categorized into three distinct developmental phases.

During Phase I (2007–2012), research production was characterized by low-volume production, with annual publications remaining in the single digits in most years, cumulatively yielding 46 papers and representing only 3.61% of the entire dataset. Bibliometric mapping identified that the earliest landmark citation burst within this domain belonged to the keyword “digestive system diseases” (2007–2017, burst strength = 3.03). This initial thematic focus precisely corresponds to the historical clinical window wherein the epidemiology of hvKP was being progressively redefined. Although KP was traditionally recognized as an opportunistic nosocomial pathogen that normalizes as a harmless inhabitant of the human gastrointestinal tract, the global dissemination of the hypervirulent pathotype since its early documentation in the 1980s and 1990s in the Asia-Pacific region shifted the clinical paradigm (Shon et al., 2013; Lee et al., 2017). Clinical and pathological investigations established that hvKP strains frequently originate from gastrointestinal carriage reservoirs. These specialized variants possess the capacity to translocate across the intestinal epithelium via transcellular cell invasion mechanisms (Odongo et al., 2022). Thereby initiating severe, systemic infections in healthy, immunocompetent individuals who completely lack traditional hepatobiliary risk factors (Eng and Hochster, 2021). Consequently, the primary clinical presentations during this era were heavily dominated by monomicrobial intra-abdominal pyogenic infections, most characteristically community-acquired pyogenic liver abscesses and concurrent splenic abscesses (Abhinand et al., 2025). During this baseline phase, because these emerging invasive hvKP strains remained highly susceptible to commonly used first-line chemical antibiotics (Russo and Marr, 2019), the therapeutic pressure to discover alternative antimicrobial agents was not yet acute. As a result, phage applications remained restricted to single-digit, sporadic *in vitro* characterizations of baseline lytic properties, keeping the annual publication volume low.

Beginning in 2013, Phase II demonstrated a progressive numerical expansion, with the annual document count steadily climbing from 15 in 2013 to 34 in 2018. The primary macromorphological inflection point within this stage is marked by the strongest citation burst intensity tracked across the entire keyword matrix, belonging to the term “*enterobacteriaceae*” (2016–2020, burst strength = 5.32). This prominent bibliometric indicator directly reflects the global public health crisis triggered by the rapid escalation of multi-drug resistant, extensively drug-resistant, and carbapenem-resistant *Enterobacteriaceae*. Within this cohort of high-risk ESKAPE pathogens, KP exhibited an extraordinary genetic flexibility. Strains actively acquired and accumulated horizontal mobile elements encoding extended-spectrum β-lactamases and diverse carbapenemases constructing a multidimensional enzymatic resistance network (Jánvári et al., 2014; Seiffert et al., 2014; Antinori et al., 2020; Biedrzycka et al., 2021; de Barsy et al., 2021). Surveillance data from this era confirm that the clinical detection rate of carbapenem-resistant *Enterobacteriaceae* continuously increased, and the incidence of healthcare-associated CRKP bloodstream infections escalated dramatically (Liu et al., 2022; Lee et al., 2023). Faced with the fatal outbreaks and intra-ward dissemination of high-risk epidemic clonal lineages within intensive care units, conventional chemical therapeutics witnessed a severe erosion of efficacy (Liu et al., 2022; Lee et al., 2023). This critical therapeutic stalemate compelled global researchers to revitalize phages as targeted, non-traditional biological weapons capable of bypassing established antibiotic resistance mechanisms (Yao et al., 2025), driving the annual publication frequency into a steady expansion phase.

Following 2019, the publication velocity underwent an extraordinary acceleration, with the annual document count escalating from 53 in 2019 to 247 in 2025. Regarding the latest temporal indicators, the contemporary research frontier is strictly bounded by the active citation bursts for “phage resistance” (2022–2026, burst strength = 3.34) and “lytic activity” (2023–2026, burst strength = 2.77). This structural shift reveals that as phage therapy successfully transitioned from experimental benchtop modeling to active clinical trials and personalized compassionate use frameworks (Chanishvili, 2012), the field encountered its most formidable biological hurdle: the rapid evolution of bacterial resistance. Because bacteriophages and bacteria exist in a continuous, reciprocal evolutionary arms race, KP undergoes rapid adaptive modifications under intense phage selective pressure. Co-evolutionary genetic analysis has delineated that adsorption blocking constitutes the primary bacterial defense strategy (Yang et al., 2025). Mutations primarily concentrate on surface polysaccharide structures, with 30.3% of documented resistance mechanisms mapping to CPS biogenesis loci and 21.4% mapping to lipopolysaccharide (LPS) core or lipid A pathways (Xing et al., 2025b). Clinical isolates frequently acquire high-level resistance via single-base deletions or insertional inactivation within the *wcaJ* gene (Sheu et al., 2023), which aborts CPS initialization, diminishes the phage’s capacity for initial receptor adsorption, and precipitates therapeutic failure via bacterial regrowth. Crucially, the cross-disciplinary integration of AI, machine learning, and deep biological foundation models, such as Evo2, has revolutionized this domain (Boeckaerts et al., 2024). Advanced neural network algorithms can now extract high-dimensional genomic features directly from whole-genome sequencing data, utilizing surface polysaccharide traits to achieve precise, strain-level prediction of phage-host specificity (Boeckaerts et al., 2024). This computational optimization removes the time-consuming bottlenecks of manual screening, unleashing unprecedented academic vitality and positioning the field for its projected peak output in 2026.

The bibliometric results of this study show that global scientific production in the field of phage applications against *Klebsiella pneumoniae* infections exhibits a clear tri-regional concentration pattern clustered across Asia, North America, and Europe. Among the 1,275 valid records included, the top 10 most productive nations contributed a cumulative volume of 50.51% of the global research output. Notably, China ranked first in research productivity with 242 documents (accounting for 18.98% of the global total), followed closely by the United States (*n* = 80, 6.27%) and India (*n* = 75, 5.88%) as the second and third most prolific contributors, respectively. The absolute advantage of Asian nations in total publication volume is inextricably linked to the global epidemiological outbreaks of hvKP and multidrug-resistant hypervirulent convergent strains, particularly within Asian regions. Multiple systematic reviews indicate that the vast majority of multidrug-resistant mutant isolations and reports on phage resistance evolution are predominantly concentrated in China (Shon et al., 2013; Lee et al., 2017; Xing et al., 2025b). Concurrently, meta-analysis has also verified that the Asian region serves as a core endemic area for hypervirulent strains triggering multi-site invasive abscesses (Khoshnood et al., 2025).

Russia exhibits a unique topological anomaly characterized by an extremely elevated citation density (CPP = 179.39) but a very low network integration value (TLS = 3). An explicit outlier analysis reveals that this profile is dually driven by macro-algorithmic data aggregation and localized high-impact clinical traditions. Globally, the total citations (*N* = 5,561) assigned to Russia within the full co-authorship network are amplified by its inclusion in highly-cited, mega-collaborative international consortia, including the landmark Lancet 2024 global antimicrobial resistance burden study (GBD 2021 Antimicrobial Resistance Collaborators, 2024), where global citations are fully accredited to all co-authoring nations. Conversely, in Russia’s independent targeted phage publication matrix, a typical long-tailed distribution is observed, demonstrating a robust Median CPP of 10.5 and an Interquartile Range of 34.5. Highly-cited outlier studies account for 51.41% (347/675) of Russia’s targeted citation pool, led by landmark papers such as Rubalskii et al. (2020) (118 citations) in cardiothoracic surgery interventions and Antonova et al. (2019) (73 citations) in broad-spectrum Myoviridae lysin engineering. These quantitative outliers directly reflect Russia’s century-long medical-historical memory and specialized clinical tradition in industrializing phage biologicals, allowing their focal translational research to garner extensive international citations despite structural isolation from Western co-authorship networks. Furthermore, the international cross-border collaboration network demonstrates that China (TLS = 48), the United Kingdom (TLS = 41), and the United States (TLS = 41) function as the primary topological hubs of the global network, while Germany (TLS = 30) and Australia (TLS = 26) serve as intermediate bridging paths to form a tight Western-hemisphere collaborative route. This directly reflects that the mainstream Western medical community has rapidly intensified its cross-border attention toward non-traditional alternative therapies like phages in recent years when facing the severe stalemate of comprehensive antibiotic failure (Yao et al., 2025).

From the institutional landscape perspective, the top 10 most productive organizations contributed a cumulative volume of 15.84% of the global total, with Chinese institutions constituting half of the core cohort. Chinese Academy of Sciencess emerged as the most prolific institution globally with 49 documents, which, together with Shanghai Jiao Tong University, Fudan University, and Zhejiang University, forms the primary research cluster in China tackling the intra-ward cross-transmission of high-risk clonal lineage. This directly maps to the grim clinical reality where the clinical detection rate of CRKP has escalated annually in Chinese hospitals, leading to a comprehensive crisis of front-line clinical therapeutics, as well as the urgency for the academic community to conduct mechanism-driven research against multidrug-resistant multidimensional regulatory networks (Zhang et al., 2018).

In contrast, Panjab University in India ranked as the second most productive institution globally with 26 documents. The pronounced academic attention garnered by this organization primarily stems from its research team’s long-standing, systematic pioneering work exploring combinations of phages or their depolymerases with chemical adjuvants (Chhibber et al., 2015). This pathway successfully counteracts the common bottleneck of biofilm-mediated treatment resistance in multi-species deep biofilms by degrading the external extracellular polymeric substance matrix. Additionally, the University of Wroctaw in Poland also ranked prominently among the leading organizations, deeply validating Poland’s outstanding academic reputation and historical contributions as a European and global backbone in the clinical standardized application of traditional phage repositories for magistral preparations (Gibson et al., 2019; Anand et al., 2020; Yerushalmy et al., 2020; Lin et al., 2021; Nagel et al., 2022).

### 4.2 Scholarly agents and dissemination pathways

Micro-mapping of the core investigator cohort demonstrates that Chinese scholars Jianqiang Li (*n* = 30) and Ying-Ying Liu (*n* = 27) lead global publication totals, functioning as primary topological hubs with individual TLS metrics of 13 and 17, respectively. In terms of academic citations and actual intellectual influence, scholar Yangheng Zhang (*n* = 17) and Jin-Town Wang (*n* = 23) from National Taiwan University tie for first place globally, each accumulating exactly 305 total citations. Concurrently, Indian scholar Sanjay Chhibber (*n* = 27) has also maintained an exceptionally prolific scientific output. This research group represents the unique paradigm of South Asian scientific forces in combating treatment-derived resistance: on one hand, actively exploring the evolutionary game between phage-induced bacterial surface polysaccharide variations and chemical adjuvants (Chhibber et al., 2015); on the other hand, actively contributing to genetic engineering and reverse vaccinology by targeting specific KP O-antigen conjugate complexes to develop novel multivalent subunits and bioconjugate vaccines, marking a significant cross-disciplinary contribution to proactive preventive strategies (Chhibber et al., 2004).

The mapping of publishing vehicles and knowledge communication channels captures a non-linear divergence between absolute publication volume and actual academic influence within the monitored domain. Frontiers in Microbiology (*n* = 55) and Scientific Reports (*n* = 27) ranked as the top two most prolific journals, with Frontiers in Microbiology also exhibiting the highest network connectivity (TLS = 237), which firmly establishes its position as the primary central intellectual hub of global knowledge dissemination. However, within the top 10 cohort, Frontiers in Cellular and Infection Microbiology achieved the highest citation density (CPP = 25.14). This pronounced capacity for hosting landmark scientific contributions is tightly coupled with the contemporary paradigms of this stage. As multi-drug resistant and hvKP lineages continuously converge globally, specialized cellular-level microbiologic insights have become crucial milestones, such as host-pathogen immune cross-talk (Huang et al., 2022; Mina et al., 2024), cellular targets (Mancuso et al., 2001; Moore et al., 2003), and multi-component anti-virulence mechanisms (Majkowska-Skrobek et al., 2018; Hu et al., 2024).

The structural topology of the global reference co-citation matrix effectively reflects the foundational knowledge blocks and thematic evolution underpinning KP phage research. Primarily, the field is anchored by a solid pathogenic foundation cluster, wherein the classical review by Paczosa and Mecsas (2016) (Microbiol Mol Biol Rev) emerged as the most prominent baseline node with 94 co-citations and an intensive network integration value (TLS = 308) (Paczosa and Mecsas, 2016). This landmark document functions as the programmatic anchor of the domain by providing a comprehensive, systematic deconstruction of cKP defense networks and virulence traits, which are crucial for bacterial colonization and systemic dissemination across divergent host tissue sites. Furthermore, this epidemiological mapping is chronologically cross-linked with the historical baseline established by Podschun and Ullmann (1998) (Clin Microbiol Rev) (Podschun and Ullmann, 1998), which originally defined the nosocomial risk structures and multi-locus pathogenicity factors of *Klebsiella* species before the widespread global emergence of hypervirulent lineages.

Transitioning from baseline pathology to genomic methods, the reference matrix captures a highly specialized bioinformatic pipeline sub-network represented by Bankevich et al. (2012) (J Comput Biol, *n* = 88, TLS = 272) (Bankevich et al., 2012) and Seemann (2014) (Bioinformatics, *n* = 68, TLS = 158) (Seemann, 2014). These nodes introduced the SPAdes de novo genome assembly algorithm and the Prokka rapid prokaryotic genome annotation tool, respectively. Although these computational methodologies accumulated massive co-citation volumes due to the routine execution of whole-genome sequencing for novel lytic phages, their topological positioning within the global network remains localized. This indicates that automated genomic sequence extraction and prokaryotic feature classification function as a distinct technical sub-thematic pipeline rather than an integrative thematic path for clinical treatment validation (Doud et al., 2025).

Concurrently, the active therapeutic dimensions of the network are dynamically interconnected by a tight cluster of breakthrough biological discoveries defining a tailspike and depolymerase core matrix, led by Lin et al. (2014) (J Infect Dis, *n* = 75, TLS = 308) (Lin et al., 2014) and Pan et al. (2017) (J Virol, *n* = 72, TLS = 317) (Pan et al., 2017). These two structural hubs pioneered the isolation, genomic architecture, and therapeutic verification of novel capsule-specific lytic phages and their virion-associated depolymerases. These foundational works successfully established the mechanical paradigm wherein phage-borne tailspike depolymerases recognize, bind, and enzymatically degrade complex host polysaccharide barrier (Lin et al., 2014; Hsieh et al., 2017; Pan et al., 2017; Wu et al., 2019). This enzymatic degradation effectively strips the anti-phagocytic shield of the pathogen, transforming phage elements from individual bactericidal agents into multi-target anti-virulence and anti-biofilm compound strategies (Majkowska-Skrobek et al., 2016; Majkowska-Skrobek et al., 2018).

Ultimately, the synchronous alignment between these high-impact biological nodes and the contemporary citation bursts underscores a significant paradigm shift toward the modern clinical and resistance frontier driven by evolutionary containment. This contemporary transition is anchored by Kortright et al. (2019) (Cell Host Microbe, *n* = 56) (Kortright et al., 2019) and Eskenazi et al. (2022) (Nat Commun, *n* = 46) (Eskenazi et al., 2022). These recent landmark contributions bridge the foundational microbiology of Klebsiella with the modern clinical landscape, where pre-adapted parallel cocktails and evolutionary phage steering are aggressively deployed to proactively counteract treatment-derived genetic resistance and restore antibiotic susceptibility during *in vivo* replication.

### 4.3 Intellectual foundations and research frontiers

The systemic co-occurrence and spatial clustering topology of Keywords Plus effectively delineate the conceptual boundaries and shifting thematic paradigms within the global landscape of KP phage research. As mapped across the density and network configurations, the intellectual domain partitions into twelve structurally distinct modular clusters that cross-link clinical danger flags with biological countermeasures, prominently featuring Cluster 0 (capsule depolymerase), Cluster 1 (phage therapy), Cluster 3 (capsular polysaccharide), Cluster 4 (phage cocktail), and Cluster 9 (carbapenem-resistant *klebsiella pneumoniae*) (Figure 9A). Within this spatial topology, the highly aggregated green cluster anchored by core treatment and formulation descriptors establishes intensive, cross-cluster connectivity with the extensive link tracks of the blue cluster containing clinical risk phenotypes like antibiotic resistance, virulence, and infection (Figure 8A). This structural configuration outlines a clear historical baseline where the domain systematically progressed from basic phenotypic characterizations of isolated viral entities toward the strategic deployment of multimodal combination therapeutics designed to dismantle complex macromolecular defenses, such as extracellular polymeric substance matrices in mature biofilms (Limoli et al., 2015; Sachdeva et al., 2017), which traditionally shelter pathogens from chemical antimicrobials.

This evolutionary trajectory is chronologically unpacked by the temporal gradient network, which reveals a non-linear paradigm shift across distinct chronological layers (Figure 8B). The chronological upstream layer is dominated by keywords displaying the lowest average publication years, which are rendered in dark blue and purple gradients. This temporal segment incorporates nodes such as mice, respiratory infection, pyogenic liver abscess, *Listeria monocytogenes*, and wound and soft tissue infection. Topologically, these earlier nodes exhibit lower cumulative link weights and a more decentralized distribution compared to the core structures of the network. This structural profile reflects an early era heavily focused on exploratory bioinformatic engineering, raw genomic mapping, and the basic characterization of localized tissue tropisms. Historically, initial research was predominantly occupied with deciphering basic genomic architectures using baseline assembly and prokaryotic characterization routines, while KP was principally investigated as a gastrointestinal symbiont capable of translocating across epithelial barriers to trigger community-acquired primary pyogenic liver abscesses and severe visceral infections (Kocsis, 2023). During this baseline interval, because these wild-type invasive pathotypes remained highly vulnerable to conventional frontline antibiotics, the wider clinical translation value of phages as therapeutic alternatives remained largely unrecognized.

Driven by intensifying public health pressures, the keyword network experienced a critical structural densification, shifting into the intermediate chronological layer which consists of terms with average publication years transitioning through green gradients between 2020 and 2021 (Figure 8B). Prominent nodes within this phase include biofilm, antibiotic resistance, phage, and therapy, which exhibit a dramatic enlargement in node size and maintain highly integrated link pathways that bridge multiple thematic clusters. This sudden surge in network connectivity directly mirrors the worldwide clinical crisis surrounding the malignant dissemination of CRKP and its convergence with hypervirulent lineages (Lei et al., 2024) across healthcare institutions. Faced with high-risk epidemic clonal lineages that rendered conventional β-lactams, carbapenems, and even last-resort chemical options ineffective, the global scientific community was forced to look backward and revitalize phages as highly targeted biological weapons capable of utilizing virion-associated depolymerases to actively dissolve outer capsule structures and liquefy deep biofilm matrices (Chen T. et al., 2024).

The contemporary research frontier is represented by the chronological downstream layer, where keywords exhibit the highest recency values up to 2023 and are highlighted in vivid yellow (Figure 8B). This cohort includes emerging nodes such as tailspike, capsule, capsular polysaccharide, cocktail, and alignment, which display prominent link tracks that intersect with established intermediate nodes to demonstrate a specialized structural convergence within the latest temporal layer. This structural transition is further corroborated by the chronological patterns of specific keyword variations detailed in the citation burst matrix spanning from 2007 to 2026 (Figure 9B). The earliest citation burst recorded within the dataset belongs to the keyword digestive system diseases, which initiated its burst interval in 2007 and concluded in 2017 with an objective burst strength of 3.03, capturing the initial baseline focus on gastrointestinal reservoirs. In terms of statistical magnitude, the maximum citation burst intensity tracked across the entire matrix is exhibited by the term *enterobacteriaceae*, which achieved a peak strength value of 5.32 during a 4-year burst period from 2016 to 2020, charting the acute era of global antibiotic resistance outbreaks. Regarding the latest temporal indicators, the most recent burst onsets are identified for the keywords “phage resistance” and “lytic activity.” The term phage resistance commenced its burst phase in 2022 with a strength of 3.34, while lytic activity initiated its burst period in 2023 with a strength of 2.77, with both signals remaining actively sustained up to the final analytical boundary of 2026.

The sustained prominent burst signals for phage resistance and lytic activity up to the present day forcefully introduce the paramount biological bottleneck currently challenging translational medicine: the rapid mutational escape of KP under intense viral selective predation (Chen H. et al., 2024). When exposed to lytic phages in clinical settings or compassionate use frameworks, clinical isolates frequently acquire treatment-derived resistance by undergoing single-base deletions or insertional inactivation within core capsular biogenesis loci, which completely blocks initial virion adsorption and triggers rapid bacterial regrowth (Cai et al., 2019; Tan et al., 2020). To preemptively neutralize this mutational evasion and secure sustainable lytic capacity, the newest yellow-coded terminal nodes (tailspike, capsule, and alignment) in close structural resonance with Cluster 0 (capsule depolymerase) and Cluster 8 (host range) introduce a revolutionary paradigm shift toward the definitive modern frontier of “AI-Driven Genomic Engineering.” Advanced machine-learning architectures and deep biological foundation models, such as Evo (Nguyen et al., 2024), are now being deployed to extract high-dimensional genomic features from whole-genome sequencing datasets to predict phage-host specificity at a strain level, completely bypassing manual screening and enabling the de novo design of synthetic phages with programmable multi-valent depolymerases to permanently guarantee sustainable lytic activity against pandrug-resistant infections (Doud et al., 2025).

### 4.4 Bacterial resistance to phages

Bacterial resistance to phages is fundamentally driven by reciprocal co-evolutionary dynamics, wherein intense selective pressure prompts random bacterial mutations and subsequent directional expansion to survive continuous viral predation (Murtazalieva et al., 2024). In clinical settings, this evolutionary pressure manifests as treatment-acquired resistance; the targeted eradication of susceptible wild-type strains allows resistant sub-populations to dynamically proliferate, ultimately leading to bacterial resurgence and therapeutic reversal. Viewed through the lens of population genetics, this resistance relies on a spontaneous mutational baseline, meaning that these mutations are random and pre-exist at low frequencies within large bacterial populations rather than being directionally induced by phages (Luria and Delbrück, 1943; Rohde et al., 2018). Furthermore, the emergence velocity of such resistance is highly sensitive to environmental modulation, varying significantly based on the surrounding microenvironment and the initial bacterial inoculum density (Nobrega et al., 2018; Kortright et al., 2019).

To counter viral threats, bacteria deploy multi-layered resistance mechanisms, beginning with adsorption blocking, which prevents the initial attachment of tailed phages to host receptors. This is primarily achieved through CPS receptor modification, where spontaneous alterations in capsule architecture abort phage binding, such as *wcaJ* gene deletions (Geng et al., 2023) or IS (GBD 2021 Antimicrobial Resistance Collaborators, 2024) KPn26 transposon insertions (Venturini et al., 2020). Similarly, LPS receptor variation involves phase variation or direct mutations in biosynthesis genes like *wecA* (Venturini et al., 2020; Hao et al., 2021), *waaC* (Hesse et al., 2020; Chen Q. et al., 2024; Yoo et al., 2024), and *fabF* (Li et al., 2023) to eliminate vital structural targets for non-contract phage. Bacteria also utilize outer membrane protein deletion and mutation, where structural defects in porins or iron transporters like *ompC* (Cai et al., 2022) and *fhuA* (Hesse et al., 2020) severely reduce adsorption efficiency. Beyond genetic loss, cells employ masking proteins and outer membrane vesicles as decoys, utilizing proteins like *TraT* to physically block receptors or hyper-secreting host-receptor-bearing outer membrane vesicles to neutralize phages before they contact the cell (Augustyniak et al., 2022; Xuan and Xuan, 2024). Finally, the upregulation of the extracellular matrix via the hyper-synthesis of extracellular polysaccharides and eDNA physically buries surface receptors, isolating the bacterial community behind a physical barrier.

When phages successfully breach surface defenses and inject their genetic material, bacteria activate intracellular nucleic acid interference and degradation systems. The classic line of defense is the restriction-modification system, a dual-enzyme mechanism that protects host DNA via methylation while employing restriction endonucleases to cleave unmethylated, non-self invading genomes (Gao et al., 2020). For adaptive defense, prokaryotes rely on CRISPR-Cas adaptive immunity, which neutralizes threats through a sequential three-step cascade consisting of spacer acquisition, crRNA expression, and Cas-mediated targeted cleavage of re-invading phage DNA (Li et al., 2025). Additionally, bacteria harbor novel multidimensional interference islands, which include non-classical clusters like the DISARM (Ofir et al., 2018), BREX (Goldfarb et al., 2015), and Argonaute systems (Swarts et al., 2014; Doron et al., 2018) that effectively block foreign DNA replication or structural configuration without necessarily inducing double-strand breaks.

If intracellular defenses fail to halt viral replication, bacteria can trigger abortive infection and cell suicide mechanisms to provide altruistic population-level protection. Toxin-antitoxin systems exemplify this paradigm by initiating programmed metabolic arrest, where host protease-mediated degradation of the unstable antitoxin liberates active toxins to stall the cell or induce premature lysis (Jurėnas et al., 2022). This is complemented by reverse transcription defense axes, such as the *DRT2* system expressing lethal Neo proteins or Retrons inducing NAD^+^ depletion, both of which trigger rapid cell death or block virion assembly upon detecting viral components (Wilkinson et al., 2024). Similarly, atypical nucleotide signaling networks, like the Kongming system (Zeng et al., 2025), are activated directly by viral triggers; here, phage-derived deoxyribonucleoside kinase prompts a KomA-KomB-KomC cascade that causes a catastrophic collapse of intracellular NAD^+^ aborting phage replication by terminating the host cell.

Lastly, bacteria coordinate atypical resistance and complex community defense strategies at both the single-cell and macro-community levels. Within biofilms, they display a sophisticated tail-fiber community defense: peripheral cells lysed by phages release a massive quantity of unassembled, free tail-fiber proteins that diffuse inward, enzymatically stripping CPS receptors from uninfected inner cells to create a protective, low-capsule zone (Hu et al., 2024). Furthermore, latent period prolongation mutations in non-receptor loci, such as *mnmE* or *rpoN* (Nang et al., 2024), alter translation or transcription efficiency to drastically delay viral release without modifying physical adsorption. Finally, superinfection exclusion and lysogenization demonstrate phage-derived defense flexibility, where non-lysogenic bacteria undergo lysogenic conversion, allowing integrated prophages to express membrane-bound proteins that selectively block the DNA injection machinery of incoming lytic phages (Crain et al., 2008).

### 4.5 AI-driven genomic engineering

The integration of AI and synthetic biology has inaugurated a paradigm shift in the management of CRKP, moving the field toward an evolutionarily informed precision medicine paradigm; however, its current implementation remains fundamentally characterized by *in silico* and preclinical exploration rather than routine bed-side translation (Doud et al., 2025; Getz et al., 2025; Wu et al., 2025). As the global burden of AMR escalates, with projections estimating 10 million annual deaths by 2050 (Saeed et al., 2023; Sawa et al., 2024), AI provides the computational power necessary to decode the vast genomic dark matter of phages, where approximately 65% of genes currently lack functional annotation (Hatfull, 2015; Wu et al., 2025). At this initial computational mining stage, breakthroughs in machine learning and deep learning frameworks, such as PHANOTATE and PhageScanner, have significantly enhanced the laboratory-scale identification of short, non-canonical open reading frames and lytic-lysogenic modules within complex metagenomic datasets (McNair et al., 2019; Albin et al., 2024; Wu et al., 2025). Furthermore, self-supervised representation learning through protein language models now allows for the annotation of viral sequence diversity, although these advanced modeling tools are currently confined to preliminary bioinformatic screening pipelines rather than real-time clinical diagnostics (Concha-Eloko et al., 2024; Flamholz et al., 2024).

Regarding structural and functional modeling, the advent of structural prediction platforms like AlphaFold2 and ESMFold has been particularly transformative for enabling residue-level engineering in silico, permitting the rational design of receptor-binding proteins and lytic enzymes prior to wet-lab synthesis (Jumper et al., 2021; Wu et al., 2025). For KP, where polysaccharide diversity is the primary host-range barrier, AI-guided domain delineation tools like DepoScope allow for the precise identification of enzymatic domains within tail fibers, facilitating rational design strategies such as domain swapping to retarget phages toward specific capsular serotypes (Yehl et al., 2019; Lenneman et al., 2021; Wu et al., 2025). Furthermore, predictive algorithms like PhageHostLearn and GSPHI now achieve up to 87% accuracy in matching phages to specific bacterial strains based on whole-genome sequences. Crucially, it must be underscored that these predictive algorithms currently function as computational benchmarking mechanisms that supplement, rather than fully substitute for, the 3–5 day delays typical of standard phenotypic assays in active clinical workflows (Pan et al., 2023; Boeckaerts et al., 2024).

Genomic engineering, primarily driven by CRISPR-Cas technology, serves as the operational arm for translating these *in silico* insights into viable preclinical constructs (Ando et al., 2015; Getz et al., 2025; Wu et al., 2025). By partitioning phage genomes into modular functional cassettes, researchers can arm synthetic phages with programmable payloads, such as sequence-specific nucleases that excise resistance genes or engineered depolymerase-lysin fusions, with efficacy currently verified *in vitro* or within animal infection models (Lu and Collins, 2007; Zhang et al., 2024). Advanced assembly platforms like VersaTile and SNIPR001 demonstrate the baseline feasibility of creating designer virions that selectively eradicate multidrug-resistant pathogens while preserving the commensal microbiome (Yosef et al., 2015; Gencay et al., 2024). While the clinical vision of an on-demand “phage printer” remains a distinctly futuristic long-term goal, the laboratory convergence of cell-free transcription-translation systems and generative genomic models is already recapitulating the basic architectures required for decentralized, point-of-care production (Rustad et al., 2018; Doud et al., 2025; Getz et al., 2025). Maintaining this rigorous separation between immediate laboratory workflows and long-term clinical potential is vital as the trajectory transitions toward evolutionarily informed precision medicine in the post-antibiotic era (Ando et al., 2015; Doud et al., 2025).

## 5 Limitations

First, although this study integrated WoSCC, Scopus, and PubMed, it omits platforms like Embase and the Cochrane Library. This limitation is standard and acceptable for bibliometric analysis. Software tools like VOSviewer and CiteSpace rely strictly on reference co-citation matrices to construct maps; the Cochrane Library focuses on clinical trials and lacks these records, while Embase metadata is structurally incompatible with direct conversion. Forcing a multi-database synthesis would cause severe metadata loss, semantic distortion, and deduplication errors.

Second, limiting data to original research articles excludes reviews and consensus guidelines, omitting broader theoretical frameworks. Restricting the analysis to English-language publications introduces a language bias, potentially underrepresenting clinical findings from regions with deep phage therapy traditions, such as Russia and Georgia, where domestic journals are heavily utilized. Additionally, including data up to May 14, 2026, introduces a temporal citation lag, preventing recent papers from reaching their true citation peaks and link strengths.

Third, the study is inherently subject to citation bias and the Matthew effect. This phenomenon drives a cumulative advantage where prominent authors or affluent nations accrue a disproportionate share of citations regardless of individual study quality. Consequently, historical research hubs may be artificially overrepresented while emerging scientific nodes are marginalized.

Fourth, caution is required when cross-interpreting results due to the different algorithmic assumptions of VOSviewer and CiteSpace. VOSviewer maps static similarity based on association strength and distance layouts, whereas CiteSpace tracks temporal evolutionary dynamics via time-slicing, log-likelihood ratios, and betweenness centrality. Because these mathematical engines use divergent boundaries, the resulting clusters may show spatial variations. Thus, these visualization outputs represent computational approximations of the field’s evolution rather than absolute clinical milestones.

## 6 Conclusion

By deploying bibliometric instruments including VOSviewer and CiteSpace, this study systematically delineates the academic landscape of bacteriophage applications against KP infections from 2007 to 2026, deeply revealing the evolutionary trajectories and collaborative dynamics of various academic entities. The findings demonstrate a clear technology-driven, exponential growth paradigm leap across three distinctive chronological stages: a baseline low-volume interval, a progressive expansion phase, and a rapid acceleration era. Throughout this evolution, the knowledge boundaries have profoundly shifted from early host-range descriptions and basic biological identification toward precision mechanistic characterization and genomic engineering driven by capsular depolymerases, tailspike proteins, and multi-omics integrations. The global distribution of scientific forces presents a differentiated yet complementary configuration: China holds absolute dominance in publication volume, with the Chinese Academy of Sciences and investigator Jianqiang Li demonstrating extraordinary productivity; however, in terms of CPP and core network integration, scholars from Europe, North America, and Oceania exhibit highly concentrated and superior academic impact. In the publishing ecosystem, Frontiers in Microbiology serves as the primary intellectual hub facilitating global knowledge exchange. From the perspective of multidrug clustering and citation bursts, the research frontier is undergoing a profound paradigm shift, where 12 core timeline clusters chart the field’s trajectory from single biological agents to complex, resistance-targeted therapeutics.

In summary, remarkable progress has been achieved globally in harnessing bacteriophages to combat *Klebsiella pneumoniae* infections, showcasing their immense potential as an innovative antimicrobial modality. Nevertheless, navigating the path toward high-efficiency clinical translation and routine practice reveals three core bottlenecks: first, the causal chains governing the dynamic resistance mechanisms between phages and host KP require deeper validation; second, rational engineering designs targeting heterogeneous capsular polysaccharides via depolymerases and tailspike proteins still lack standardized algorithmic support; third, bounded by database retrieval constraints and temporal citation lags, a global scarcity of large-scale, multicenter clinical efficacy evidence remains evident. Consequently, future research endeavors should be concentrated on three pivotal directions: first, integrating AI structural prediction platforms with CRISPR-Cas genomic editing technologies to achieve precise reconstruction of receptor-binding proteins and develop customized “phage cocktails”; second, deepening molecular dynamics research into the interplay between phage lytic activity and host resistance mechanisms to bypass evolutionary adaptations; and finally, strengthening cross-border, inter-institutional, and interdisciplinary collaborations to establish unified, full-chain technical standards and regulatory frameworks for phage biological products. This concerted effort will accelerate the leap of anti-KP phage therapy from benchside fundamental science to precision bedside clinical application.

## Data Availability

The raw data supporting the conclusions of this article will be made available by the authors, without undue reservation.
